# Effect of Dietary Minerals on Virulence Attributes of *Vibrio cholerae*

**DOI:** 10.3389/fmicb.2017.00911

**Published:** 2017-05-19

**Authors:** Varunkumar Bhattaram, Abhinav Upadhyay, Hsin-Bai Yin, Shankumar Mooyottu, Kumar Venkitanarayanan

**Affiliations:** ^1^Department of Animal Science, University of Connecticut, StorrsCT, United States; ^2^Department of Poultry Science, University of Arkansas, FayettevilleAR, United States

**Keywords:** *Vibrio cholerae*, virulence, adhesion, motility, cholera toxin, essential minerals, gene expression

## Abstract

*Vibrio cholerae* is a water-borne pathogen responsible for causing a toxin-mediated profuse diarrhea in humans, leading to severe dehydration and death in unattended patients. With increasing reports of antibiotic resistance in *V. cholerae*, there is a need for alternate interventional strategies for controlling cholera. A potential new strategy for treating infectious diseases involves targeting bacterial virulence rather than growth, where a pathogen’s specific mechanisms critical for causing infection in hosts are inhibited. Since bacterial motility, intestinal colonization and cholera toxin are critical components in *V. cholerae* pathogenesis, attenuating these virulence factors could potentially control cholera in humans. In this study, the efficacy of sub-inhibitory concentration (SIC, highest concentration not inhibiting bacterial growth) of essential minerals, zinc (Zn), selenium (Se), and manganese (Mn) in reducing *V. cholerae* motility and adhesion to intestinal epithelial cells (Caco-2), cholera toxin production, and toxin binding to the ganglioside receptor (GM1) was investigated. Additionally, *V. cholerae* attachment and toxin production in an *ex vivo* mouse intestine model was determined. Further, the effect of Zn, Se and Mn on *V. cholerae* virulence genes, *ctxAB* (toxin production), *fliA* (motility), *tcpA* (intestinal colonization), and *toxR* (master regulon) was determined using real-time quantitative PCR. All three minerals significantly reduced *V. cholerae* motility, adhesion to Caco-2 cells, and cholera toxin production *in vitro*, and decreased adhesion and toxin production in mouse intestine *ex vivo* (*P* < 0.05). In addition, Zn, Se, and Mn down-regulated the transcription of virulence genes, *ctxAB, fliA*, and *toxR.* Results suggest that Zn, Se, and Mn could be potentially used to reduce *V. cholerae* virulence. However, *in vivo* studies in an animal model are necessary to validate these results.

## Introduction

*Vibrio cholerae*, the causative agent of cholera, is a motile, Gram-negative, comma shaped bacterium responsible for producing profuse diarrhea in humans. Of the 150 serogroups of *V. cholerae*, O1 and O139 are important because they are responsible for causing pandemics across Asia, Africa, and Latin America (Faruque et al., 1998; Sack et al., 2006). The O1 serogroup is further subdivided into classical and El Tor biotypes. The classical biotype was responsible for six cholera pandemics before 1961, whereas the El Tor biotype was implicated in pandemics after 1961 (Ramamurthy et al., 2003).

The pathogenesis of cholera is primarily mediated by *V. cholerae* motility, followed by binding to the intestinal epithelial cells and production of cholera toxin in the intestinal lumen. Cholera toxin is an oligomeric protein complex composed of two subunits, subunit A (28 kDa) and subunit B (11 kDa). Once attached to the intestinal epithelium, *V. cholerae* releases the exotoxin, which by means of its pentameric subunit B binds to the GM1 ganglioside receptor present on the surface of intestinal epithelial cells, thereby leading to endocytosis of the toxin into the cell cytoplasm. Following the dissociation of the toxin in the endoplasmic reticulum, the monomeric subunit A causes an increase in the cytoplasmic adenylate cyclase activity leading to an ATP-mediated efflux of sodium and potassium ions in the intestinal lumen. To counter the increase in solute concentration in the lumen, the cells secrete water, and this accumulation of water in the lumen causes dehydrating diarrhea in patients affected by *V. cholerae* (Ichinose et al., 1987; Ikigai et al., 1996; Olson and Gouaux, 2005; O’Neal et al., 2005; Sánchez and Holmgren, 2011). Thus, reducing *V. cholerae* motility, intestinal attachment and toxin production could potentially control cholera in humans.

Currently, the most common treatment strategy against cholera is oral rehydration therapy (Iwu et al., 1999; Sack et al., 2006), which only restores fluids to patients and aids in recovery from dehydration. Although antibiotics are frequently administered to reduce the severity and duration of the disease, a majority of *V. cholerae* strains from cholera endemic countries have been reported to be resistant to multiple antibiotics (Garg et al., 2000; Chakraborty et al., 2001). Moreover, oral vaccines against *V. cholerae* are not completely effective in providing protective immunity (World Health Organization [WHO], 2011 Guidelines). Thus, there is a need for effective and easily implementable approaches that primarily target the pathogen for controlling cholera.

Since ancient times, metals have been known to exert antimicrobial effect against various microorganisms. Following the discovery of antibiotics in the 1920s, the use of metals as antimicrobial agents diminished rapidly in human medicine. However, with the emergence of antibiotic resistant strains of pathogens and a lack of new and effective antibiotics, interest in the use of metals as antimicrobial agents is renewed. Zinc, selenium, and manganese are naturally occurring essential microelements recommended for daily intake by the United States Food and Drug Administration. These minerals are present in a wide range of foods in addition to their presence in dietary supplements. Selenium plays structural and enzymatic roles in many biological processes in humans (Rayman, 2000; Kryukov et al., 2003). Manganese (Mn) is an essential micronutrient critical for the activity of a variety of enzymes, and is required for proper immune function, regulation of blood sugar and cellular energy, reproduction, and blood coagulation (Horning et al., 2015). Manganese (Mn) possesses potent anti-inflammatory and anti-oxidant properties (Kovala-Demertzi et al., 2009). Zinc (Zn) is important for cell division and growth, and proper functioning of the immune system. The antimicrobial property of Zn is well documented (Espitia et al., 2012; Singh et al., 2012), and its supplementation has been reported to exert a beneficial effect in controlling diarrhea in children (World Health Organization [WHO], 2009, 2011). A study conducted in Bangladesh found that zinc supplementation significantly reduced the duration of diarrhea and stool output in children with cholera (Roy et al., 2008).

The objective of this study was to investigate the effect of sub-inhibitory concentration (SIC, highest concentration that does not affect bacterial growth) of Zn, Se, and Mn in reducing *V. cholerae* motility, adhesion to intestinal epithelial cells, cholera toxin production, and toxin binding to GM1 ganglioside receptor *in vitro.* Since intestinal attachment and toxin production play a key role in the pathogenesis of cholera, an *ex vivo* mouse intestine model was also used to determine the effect of metals *V. cholerae* adhesion and cholera toxin production. In addition, the effect of Zn, Se, and Mn on the transcription of genes associated with aforementioned virulence factors was studied.

## Materials and Methods

### Bacterial Strains and Culture Conditions

All bacteriological media were purchased from Difco (Becton Dickinson, Sparks, MD, United States). Three strains of *V. cholerae*, including BAA-25870, BAA-2163, and N16961 (denoted as VC 569b, VC 2163 and VC N16961, respectively) were used. Each strain was individually cultured in sterile 10 mL tubes containing alkaline peptone broth supplemented with 0.5% sodium bicarbonate, and incubated at 37°C for 24 h (Tran et al., 2012). Following incubation, the cultures were sedimented into a pellet by centrifugation (3600 × *g* for 15 min), washed twice and resuspended in 10 mL of sterile phosphate buffered saline (PBS, pH 7.0). Serial 10-fold dilutions were plated onto Tryptic Soy Agar (TSA) and Thiosulfate Citrate Bile Salt Sucrose agar (TCBS) plates, and *V. cholerae* colonies were enumerated after incubation at 37°C for 24 h.

### SIC Determination

The SIC of Zn (Zinc chloride, Sigma–Aldrich, St. Louis, MO, United States), Se (Sodium selenite, Sigma–Aldrich) and Mn (Manganese chloride, Sigma–Aldrich) was determined, as previously described (Johny et al., 2010; Amalaradjou et al., 2011; Upadhyay et al., 2013). Briefly, ∼ 5 log *V. cholerae* was added to sterile 10 mL tubes containing alkaline peptone broth containing 0.5% Sodium bicarbonate, followed by the addition of tapering concentrations of Zn, Se, and Mn from 0.2% to 0.005% (wt/vol) in increments of 0.05%. The tubes were incubated at 37°C for 24 h, and bacteria were enumerated as described before on TSA and TCBS agar. The highest concentration of each metal that did not inhibit *V. cholerae* growth at 24 h was selected as the respective SIC for this study.

### Motility Assay

The effect of Zn, Se, and Mn on *V. cholerae* motility was determined according to a published protocol (Niu and Gilbert, 2004). Briefly, Luria Bertani (LB) agar (0.3%) containing the respective SIC of Zn, Se, and Mn was prepared. LB agar without the metals served as control. An overnight culture of *V. cholerae* was centrifuged at 3600 × *g* for 15 min and was washed three times with sterile PBS. Twenty μL of the resuspended culture (∼8 log CFU/mL) was spot inoculated at the center of the LB agar plates, incubated at 37°C for 16 h, and the zone of motility was measured (cm).

### Cell Culture

Human intestinal epithelial cells (Caco-2, ATCC-HTB37, Manassas, VA, United States) were maintained in DMEM (GIBCO, Invitrogen, Carlsbad, CA, United States) containing 10% Fetal Bovine Serum (FBS, Invitrogen). Trypsin-treated cells were passaged five times and were seeded onto 12-well cell culture plates (∼6 × 10^5^ cells per well), and grown at 37°C in the presence of 5% CO_2_ for 48 h to form a monolayer.

### Adhesion Assay

The effect of Zn, Se, and Mn on *V. cholerae* adhesion to Caco-2 was investigated by following a published protocol (Krebs and Taylor, 2011). Briefly, the cells were seeded in 24-well tissue culture plates and incubated at 37°C in a humidified, 5% CO_2_ incubator for 18 h. Overnight culture of each *V. cholerae* was washed and resuspended in Caco-2 culture medium supplemented with SIC of Zn, Se, or Mn. Approximately 6 log CFU (MOI of 10:1) of *V. cholerae* suspension was added to the monolayer either in the presence or absence of Zn, Se, and Mn. Following a 2 h incubation at 37°C, the infected monolayer was rinsed three times with sterile PBS and the cells were lysed with 0.1% Triton X-100. Viable adherent bacteria were enumerated by serial 10-fold dilution in PBS and plating on TCBS agar following incubation at 37°C for 24 h.

### Cholera Toxin Quantification

The effect of Zn, Se, and Mn on toxin production by *V. cholerae* strains was determined using Enzyme linked Immunosorbent assay (ELISA) that was customized for Cholera toxin using a published method with alterations (Upadhyay et al., 2013). Protein detector ELISA kit (Kierkegaard and Perry Laboratories, Gaithersburg, MD, United States) was standardized using pure cholera toxin (Sigma–Aldrich) and Cholera toxin b-subunit (Sigma–Aldrich). *V. cholerae* was grown for 24 h at 37°C in sterile 10 mL alkaline peptone broth with the SIC of Zn, Se, or Mn. The cultures were centrifuged at 4000 × *g* for 20 min and the supernatants were collected. Polystyrene 96-well ELISA plate (Fisher Scientific, Pittsburg, PA, United States) was coated with anti-Cholera toxin B-subunit (Sigma–Aldrich) that was diluted 1:500 in coating buffer. After 1 h incubation, the wells were washed five times with a washing buffer followed by a 15 min blocking step with 1% bovine serum albumin (BSA, Sigma–Aldrich) to prevent non-specific binding. Following the blocking step, the sterile filtered supernatant of *V. cholerae* cultures was incubated in the wells for 2 h. After washing, the wells were incubated with a HRP-conjugated antibody against the B-subunit (1:2500 dilution) of the toxin. Peroxide solution was added and the colorimetric analysis was done using a spectrophotometer at 405 nm. As a positive control, 100 ng/mL of cholera toxin was incubated in a separate well. The results were represented as ng/mL of the toxin in the supernatant based on a standard curve.

### Cholera Toxin-GM1 Binding Assay

The effect of Zn, Se, and Mn on the binding of Cholera toxin to GM1 receptor was determined using an ELISA protocol (Lindholm et al., 1983). Briefly, 0.5 μg/mL of GM1 was coated onto 96-well polystyrene plates. The wells were then blocked with 1% BSA to prevent non-specific binding. The culture free broth containing the pure toxin treated without (control) and with SIC of Zn, Se, or Mn was then added to the wells, and incubated for 2 h. Following a 5X wash step, HRP-conjugated secondary antibody was added and incubated for 1 h. Peroxide solution was subsequently added to the wells and the optical density was measured at 405 nm.

### *Ex Vivo* Mouse Intestine Adhesion and Toxin Production Assay

*Ex vivo V. cholerae* adhesion assay was performed following a published protocol (Liu et al., 2011; Ajjampur et al., 2016). Briefly, from euthanized 5-week old BL6 mice (procured from the Department of Physiology and Neurobiology, University of Connecticut), small intestine was cut open, trimmed to equal-sized pieces, and placed in 6-well dishes containing DMEM, supplemented with 10% FBS. Approximately 6 log CFU of *V. cholerae* were inoculated to wells with or without the SIC’s of Zn, Se, and Mn. Viable adherent *V. cholerae* to mouse intestine were enumerated after 24 h by homogenization and serial 10-fold dilutions and plating on TSA+1.5% agar plates followed by incubation at 37°C. In addition, cell free supernatant obtained from each well and subjected to centrifugation at 4000 × *g* for 20 min was used to estimate the toxin concentration.

### RNA Isolation and Real-Time Quantitative PCR (RT-qPCR)

The effect of Zn, Se, and Mn on the expression of *V. cholera* virulence genes was investigated using real-time quantitative PCR (RT-qPCR) (Coutard et al., 2007). Each *V. cholerae* strain was grown separately with the SIC of Zn, Se, or Mn at 37°C in alkaline peptone broth, and total RNA was extracted after 24 h using a RNeasy RNA isolation kit (Qiagen, Valencia, CA, United States). The complementary DNA (cDNA) synthesized using the IScript cDNA synthesis kit (Bio-Rad, Hercules, CA, United States) was used as the template for RT-qPCR. The amplification product was detected using Power^®^ SYBR green reagent (Bio-Rad). The primers of each gene used in this study (*ctxAB, fliA, tcpA*, and *toxR*) were designed from published *V. cholerae* sequences using NCBI Primer Blast. Relative gene expression was determined using comparative critical threshold (ct) method using a 7500 Step one real Time PCR system (Applied Biosystems, Carlsbad, CA, United States). Data were normalized to the endogenous control (16S rRNA), and the level of the candidate virulence gene expression between treated and control samples was compared by analyzing the RQ values.

### Statistical Analysis

A completely randomized design was used for the study. All experiments had duplicate samples and were repeated three times. The data from independent, replicate trials were pooled and analyzed using the PROC MIXED subroutine of the Statistical Analysis Software (SAS ver. 9.2). The least significant difference test was used to determine significant differences (*P* < 0.05) due to treatments on bacterial counts. Data comparisons for the gene expression study were made by using Student’s *t*-test. Differences were considered significant when the *P*-value was < 0.05.

## Results

### Sub-inhibitory Concentration and Effect of Zn, Se, and Mn on *V. cholerae* Motility

**FIGURE 1:**
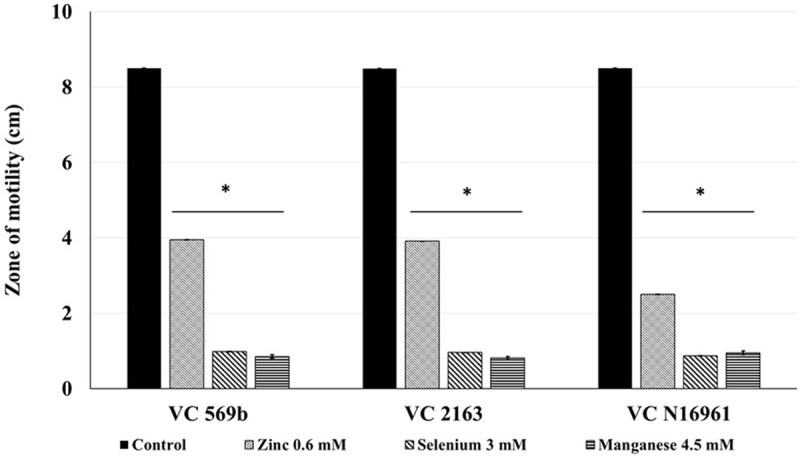
**Effect of SIC’s of Zn, Se, and Mn on motility of *Vibrio cholerae* (Strains VC 569b, VC 2163, and VC N16961).** Error bars represent SEM (*n* = 6; *P* < 0.05). ^∗^Treatment significantly different from control.

The SIC of Zn, Se, and Mn that did not inhibit the growth of *V. cholerae* strains at 24 h of incubation were 0.6, 3, and 4.5 mM, respectively. The effect of SIC of Zn, Se, and Mn on *V. cholerae* motility is shown in **Figure 1**. The average zone of motility produced by VC 569b in the absence of metals (control) was ∼8.5 cm. However, in the presence of SIC of Zn, Se, and Mn, the zone of motility was reduced to 4.0, 0.9, and 0.85 cm, respectively (*P* < 0.05). Similarly, the average zone of motility of untreated VC 2163 and VC N16961 was 8.5 cm. In the presence of Zn, the motility zone of these isolates was decreased to ∼50%, whereas Se and Mn reduced the motility by ∼90% (*P* < 0.05). Taken together, these results show that Zn, Se, and Mn have a significant inhibitory effect on *V. cholera* motility.

### Effect of SIC of Zn, Se, and Mn on *V. cholerae* Adhesion to Caco-2 Cells

**FIGURE 2:**
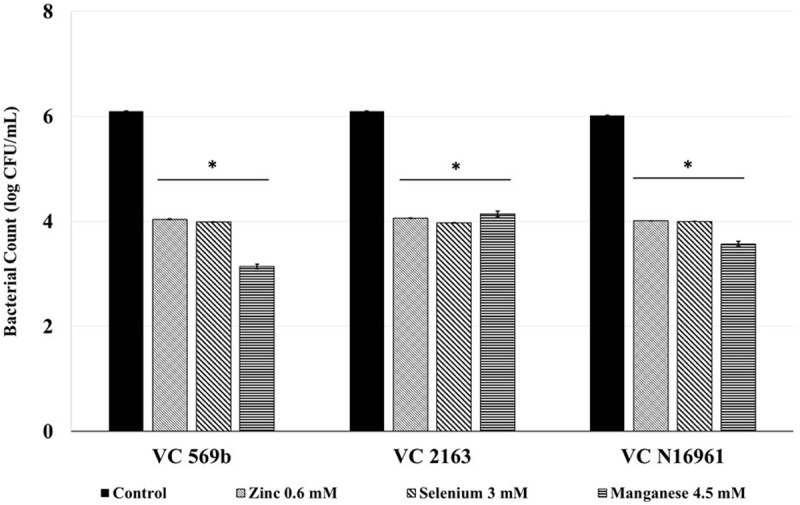
**Effect of SIC’s of Zn, Se, and Mn on *V. cholerae* adhesion (Strains VC 569b, VC 2163, and VC N16961) to Caco-2 intestinal epithelial cells.** Error bars represent SEM (*n* = 6, *P* < 0.05). ^∗^Treatment significantly different from control.

The SIC of Zn, Se, and Mn significantly reduced the number of adherent *V. cholerae* to Caco-2 cells **Figure 2**. Approximately 6.0 log CFU/mL *V. cholerae* attached to the intestinal epithelial cells in the control wells for all three strains used in this study. However, Zn, Se, and Mn decreased Caco-2 cell adhesion of all *V. cholerae* isolates by ∼2 log CFU/mL (*P* < 0.05), indicating that these minerals have a significant inhibitory effect on *V. cholerae* adhesion to intestinal epithelial cells.

### Effect of SIC of Zn, Se, and Mn on Cholera Toxin Production

**FIGURE 3:**
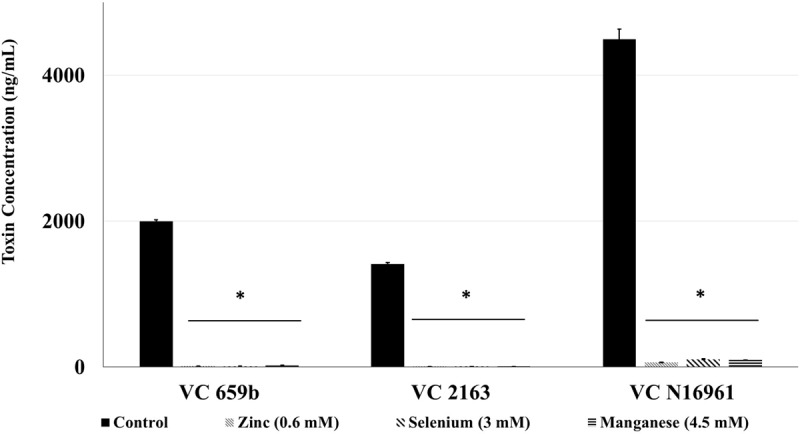
**Effect of SIC’s of Zn, Se, and Mn on cholera toxin production in *V. cholerae* (VC 569b, VC 2163, and VC N16961).** Error bars represent SEM (*n* = 6; *P* < 0.05). ^∗^Treatment significantly different from control.

The toxin concentration in the filtered supernatant was determined using ELISA, and the results are shown in **Figure 3**. In control samples, the concentration of toxin produced by the three isolates ranged from 2000 ng/mL (by VC 569b) to 4500 ng/mL (by VC N16961). However, at the respective SIC, Zn, Se, and Mn significantly reduced the toxin production in all three *V. cholerae* isolates (*P* < 0.05). For example, Zn decreased the toxin concentration to 14.5, 8.8, and 150 ng/mL in isolates 569b, 2163, and N16961, respectively. Similarly, the total concentration of the toxin detected the culture supernatant of Se-treated 569b, 2163, and N16961 were 11.8, 9.4, and 150 ng/mL and in Mn-treated culture supernatant 569b, 2163, and N16961 were 24, 7.458, and 97.25 ng/mL, respectively. Collectively, these results show that Zn, Se, and Mn reduced the production of Cholera toxin by more than 95% compared to control.

### Effect of Zn, Se, and Mn on the Binding of Cholera Toxin to GM1

Since the binding of Cholera toxin to the GM1 receptor in the human intestinal epithelial cells is a critical step *V. cholerae* pathogenesis, we investigated the effect of SIC of Zn, Se, and Mn on the binding of toxin to the receptor. Results indicate that the metals did not exert any effect on toxin binding to GM1 receptor.

### *Ex Vivo* Mouse Intestine Adhesion and Toxin Production Assay

**FIGURE 4:**
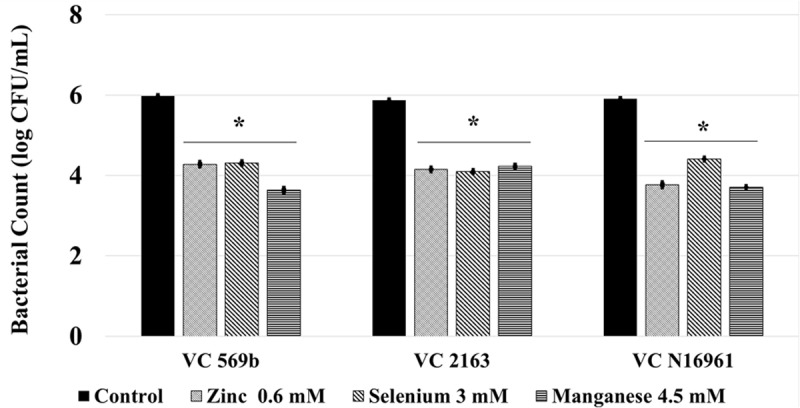
**Effect of SIC’s of Zn, Se, and Mn on *V. cholerae* adhesion to mice intestine *ex vivo*.** Error bars represent SEM (*n* = 6; *P* < 0.05). ^∗^Treatment significantly different from control.

**FIGURE 5:**
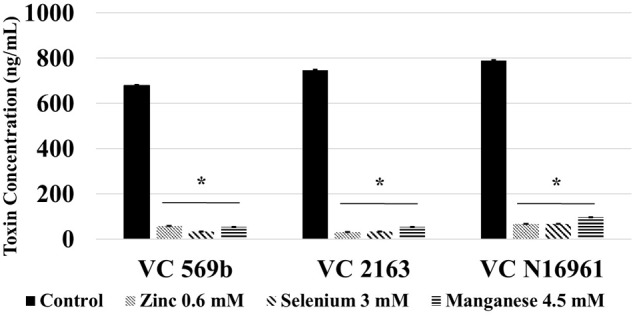
**Effect of SIC’s of Zn, Se, and Mn on cholera toxin production in *V. cholerae* in mouse intestine *ex vivo* (VC 569b, VC 2163, and VC N16961).** Error bars represent SEM (*n* = 6; *P* < 0.05). ^∗^Treatment significantly different from control.

Following a 24 h incubation period, the count of adhered *V. cholerae* in control was approximately 6.0 log CFU/mL. Compared to control, Zn, Se, and Mn (**Figure 4**) reduced bacterial attachment by ∼2.0 log CFU/mL (*P* < 0.05). Similarly, the amount of toxin produced by the three *V. cholerae* isolates in control samples ranged from 700 to 800 ng/mL. Similar to the *in vitro* results, Zn, Se, and Mn reduced toxin production by more than 90% (<100 ng/mL) in all the three strains tested (**Figure 5**).

### Effect of Zn, Se, and Mn on the Transcription of *V. cholerae* Virulence Genes

**FIGURE 6:**
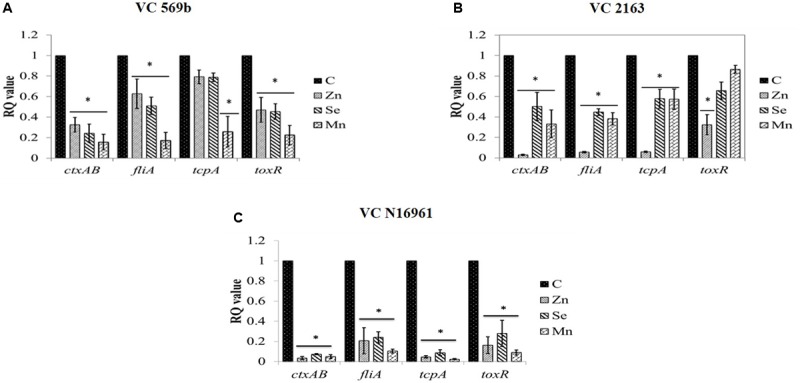
**Relative fold change in the expression level of *V. cholerae* (A)** Strains VC 569b, **(B)** VC 2163, and **(C)** VC N16961 virulence genes in response to SIC’s of Zn, Se, and Mn. ^∗^RQ value of treatment significantly different from control.

The effect of SIC of Zn, Se, and Mn on the expression of virulence genes in strains VC569b, VC 2163, and VC N16961 is shown in **Figures 6A–C**, respectively. As evident from the RQ values, Zn, Se, and Mn significantly down-regulated the expression of *ctxAB, fliA, tcpA*, and *toxR* in all three *V. cholerae* strains (*P* < 0.05) although the magnitude of reduction was greatest in VC N16961. Among the three genes tested, the down-regulation of *ctxAB* was found to be generally greater in all VC strains compared to other genes.

## Discussion

Human cholera continues to pose a significant public health concern in the developing world, with a stable increase in the number of cases reported since 2007 (World Health Organization [WHO], 2012). Currently the most common treatment strategy against cholera is oral rehydration therapy, which primarily restores fluids to patients and aids in recovery from dehydration (Guerrant et al., 2003). In addition, antibiotics are frequently administered to reduce the severity and duration of the disease (Yamasaki et al., 2011; Anthouard and DiRita, 2013). However, the development of bacterial antibiotic resistance is a concern, and a majority of *V. cholerae* strains involved in cholera endemic countries have been reported to be resistant to multiple antibiotics (Garg et al., 2000; Chakraborty et al., 2001; Das et al., 2008). This has triggered renewed interest in identifying effective alternate approaches to control cholera in humans (Fazil and Singh, 2011; Hung et al., 2005; Yamasaki et al., 2011). Treatments that target *V. cholerae* toxin production and/or colonization alone or in combination with existing therapies represent a potential strategy for controlling this pathogen (Anthouard and DiRita, 2013).

A new approach that is increasingly being explored for controlling infectious diseases involves inhibiting bacterial virulence rather than growth, where a pathogen’s specific mechanisms critical for causing infection or disease symptoms in hosts are targeted (Rasko and Sperandio, 2010; Anthouard and DiRita, 2013; Khodaverdian et al., 2013). Since anti-virulence agents are neither bacteriostatic nor bactericidal, they exert a reduced selection pressure for the development bacterial drug resistance (Hung et al., 2005; Clatworthy et al., 2007; Cegelski et al., 2008; Mellbye and Schuster, 2011; Maeda et al., 2012), besides being minimally deleterious on the host endogenous microbiota.

*Vibrio cholerae* is a highly motile organism, where motility as a first step in its pathogenesis helps the bacterium for traversing through the intestine, especially to penetrate through the mucus layer and reach the intestinal cells (Guentzel and Berry, 1975), where it colonizes. Several studies have reported motility as a virulence factor critical for *V. cholerae* colonization and pathogenesis (Guentzel and Berry, 1975; Yancey et al., 1978; Gardel and Mekalanos, 1996; Syed et al., 2009; Millet et al., 2014). The results from the motility assay revealed that Zn, Se, and Mn substantially reduced motility in *V. cholerae*, although the mechanism behind the antimotility effect of the metals is not clear. Previous studies done in *Escherichia coli* O157:H7 and *Campylobacter jejuni* propose that minerals could alter the membrane integrity and cellular morphology thereby affecting the flagellar structures, which are essential for bacterial motility (Catrenich and Makin, 1991; Adams et al., 2003). However, further studies have to be done to elucidate the exact mechanism of action.

Since bacterial attachment to the small intestinal epithelium is essential for *V. cholerae* colonization and toxin production (Krebs and Taylor, 2011), we investigated the effect of Zn, Se, and Mn on its adherence to cultured Caco-2 cells. All three minerals were found to reduce bacterial attachment to the intestinal epithelial cells in all the three tested isolates of *V. cholerae*. Further Zn, Se, and Mn were very effective in inhibiting cholera toxin production, and reduced total toxin concentration by greater than 95% in the three *V. cholerae* isolates. Concurring with the *in vitro* results, Zn, Se, and Mn reduced both *V. cholerae* adhesion and toxin production *ex vivo*. This is important since Cholera toxin produced by the bacterium constitutively activates adenylate cyclase in host cells, and leads to a decrease in sodium uptake with a concurrent increase in chloride influx into the intestinal lumen, thereby resulting in profuse diarrhea and dehydration (Sánchez and Holmgren, 2011). However, Zn, Se, and Mn were not found to affect binding of cholera toxin to GM1 receptor on the intestinal epithelial cells, as revealed by the results from binding assay. These results suggest that Zn, Se, and Mn do not exert an inhibitory effect against the toxin on the host cells, but primarily act through the virulence machinery of the bacterium.

Since the minerals were used at their respective SIC in this study, the attenuation of the *V. cholerae* virulence observed is not due to growth inhibition, but could be attributed to the effect of metals in modulating the transcription of respective bacterial genes associated with virulence. Therefore, we performed a RT-qPCR to determine the effect of Zn, Se, and Mn on major genes that are known to play a vital role in *V. cholerae* virulence in humans. Of the genes tested, *toxR* is transcription activator that controls many virulence factors, including cholera toxin, pilus colonization factor and outer membrane protein expression in *V. cholerae* (Krukonis et al., 2000; Bina et al., 2003). Similarly, *fliA* is a RNA polymerase sigma factor in *V. cholera* that controls flagella-related genes and motility in the bacterium (Guentzel and Berry, 1975), whereas *ctxAB* encodes the cholera toxin (DiRita et al., 1991). In addition, *tcpA* encodes co-regulated pilus that is responsible for intestinal colonization of *V. cholerae* (Herrington et al., 1988). Concurring with the results from phenotypic tests, it can be observed that Zn, Se and Mn exerted a significant inhibitory effect on the virulence genes in *V. cholerae* (**Figure 4**). However, the magnitude of inhibition of the genes differed in the three strains, with highest down-regulation of all the genes observed in VC N16961. Among the various tested genes, *ctxAB* was generally more sensitive to the inhibitory effect of the minerals, which concurred with the results from phenotypic tests, where cholera toxin production was found to be decreased by more than 95% in all three *V. cholerae* strains. In conclusion, this study indicated that Zn, Se, and Mn significantly reduced the major virulence properties in *V. cholerae*, especially the toxin production. The mechanism by which these minerals attenuate *V. cholerae* virulence is not known; however, emerging evidence suggests metal induced reactive oxygen species (ROS) and genotoxicity could be potential mechanisms by which metals exert antibacterial activity. For example, in enteric pathogens such as *E. coli*, production of ROS and superoxide anions leads to significant damage of DNA, membrane and cellular proteins (Imlay et al., 1988; Critchfield et al., 1993; Imlay, 2003; Parvatiyar et al., 2005; Teitzel et al., 2006; Pérez et al., 2007; Harrison et al., 2009; Warnes et al., 2012). However, transcriptomic analysis of *V. cholerae* exposed to Zn, Se, and Mn could potentially identify critical genome-wide pathways modulated by these essential minerals.

The SIC of Se at which the down-regulation of virulence observed in this study is approximately at the recommended upper tolerable levels (400 μg) and well below the no-observed-adverse-effect level (NOAEL, 800 μg/day) of Se (National Institute of Medicine, 2000). This is also the same for the other two metals, where the upper tolerable limit of Zn is 40 mg and the NOAEL is reported at 50 mg and for Mn, both the upper tolerable limit and the NOAEL have been reported at 11 mg (Scientific Committee on Food of European Commission [SCFEC], 2002; Valko et al., 2005; De et al., 2006). Since these minerals are soluble in water, they could be incorporated as ingredients in the oral rehydration solution. Follow up *in vivo* studies are needed to validate the safety and efficacy of Zn, Se, and Mn in controlling cholera.

## Author Contributions

VB, AU, SM, and KV designed the study. VB, AU, H-BY, and SM conducted the experiments. AU and H-BY analyzed the data and VB wrote the manuscript. AU and KV critically analyzed and revised the manuscript.

## Conflict of Interest Statement

The authors declare that the research was conducted in the absence of any commercial or financial relationships that could be construed as a potential conflict of interest.
